# Numerical Simulation of an Inverted Perovskite Solar Cell Using a SiOx Layer as Down-Conversion Energy Material to Improve Efficiency and Stability

**DOI:** 10.3390/ma16237445

**Published:** 2023-11-30

**Authors:** Ezequiel Paz Totolhua, Jesús Carrillo López, Alfredo Benítez Lara, Karim Monfil Leyva, Ana C. Piñón Reyes, Javier Flores-Méndez, José Alberto Luna López

**Affiliations:** 1 Centro de Investigaciones en Dispositivos Semiconductores (CIDS-ICUAP), Benemérita Universidad Autónoma de Puebla (BUAP), Col. San Manuel, Cd. Universitaria, Av. San Claudio y 14 Sur, Puebla 72570, Mexico; ezequiel.paz@alumno.buap.mx (E.P.T.); karim.monfil@correo.buap.mx (K.M.L.); javier.flores@puebla.tecnm.mx (J.F.-M.); 2 Centro de Investigaciones en Óptica, A.C. (CIO), Lomas del Bosque 115, Colonia Lomas del Campestre, León, Guanajuato 37150, Mexico; alfredbl@cio.mx; 3 Tecnológico Nacional de México/I.T. Puebla, Av. Tecnológico No. 420, Maravillas, Puebla 72220, Mexico; anacecilia.pinon@puebla.tecnm.mx

**Keywords:** inverted PSCs, simulation and optimization, down-conversion, silicon-rich oxide, power conversion efficiency, SCAPS-1D software

## Abstract

Inverted perovskite solar cells (PSCs) have gained much attention due to their low hysteresis effect, easy fabrication, and good stability. In this research, an inverted perovskite solar cell ITO/PEDOT:PSS/CH_3_NH_3_PbI_3_/PCBM/Ag structure was simulated and optimized using SCAPS-1D version 3.3.10 software. The influence on the device of parameters, including perovskite thickness, total defect density, series and shunt resistances, and operating temperature, are discussed and analyzed. With optimized parameters, the efficiency increased from 13.47% to 18.33%. Then, a new $\mathrm{Si}O_{x}$/ITO/PEDOT:PSS/CH_3_NH_3_PbI_3_/PCBM/Ag device was proposed which includes a silicon-rich oxide ($\mathrm{Si}O_{x}$) layer. This material was used as the down-conversion energy material, which converts high-energy photons (ultraviolet UV light) into low-energy photons (visible light), improving the stability and absorption of the device. Finally, with $\mathrm{Si}O_{x}$, we obtained an efficiency of 22.46% in the simulation. Therefore, the device with the $\mathrm{Si}O_{x}$ layer is the most suitable as it has better values for current density–voltage output and quantum efficiency than the device without $\mathrm{Si}O_{x}$.

## 1. Introduction

Perovskite solar cells (PSCs) have become a focus of research since they appeared in 2009 and are currently the most promising devices of the future. In just a few years, they have made significant progress, and it has been demonstrated that the power conversion efficiency (PCE) has already reached 25.5%, which is comparable to silicon (Si), gallium arsenide (GaAs) and cadmium telluride (CdTe) technology [1]. Most perovskite solar cells (PSCs) have the traditional vertical structure (n-i-p type). Nevertheless, vertical structure devices suffer from severe hysteresis effects and have poor stability. In contrast, the inverted or p-i-n type structure has good interface stability and fewer faulty states between the interfaces, and it is also possible to fabricate flexible devices at low temperatures [2].

Meanwhile, metal-organic perovskite ${(\mathrm{CH}}_{3}NH_{3}\mathrm{Pb}I_{3})\;$remains one of the materials used in perovskite solar cells due to its simple synthesis process, low cost, and high performance [3]. Nevertheless, its instability and rapid environmental degradation remain obstacles to its commercialization [4]. In addition, methylammonium lead iodide perovskite ${(\mathrm{CH}}_{3}NH_{3}\mathrm{Pb}I_{3})\;$has many advantages: high absorption coefficient, tunable band gap, high charge carrier mobilities, low trap density, long carrier recombination time and small exciton binding energy [5].

Although methylammonium lead iodide perovskite has good photovoltaic characteristics, scientists are now using other cations to replace lead (Pb). Pb is not environmentally friendly and has deleterious effects on humans and the environment. Consequently, numerous theoretical analyses have shown that inorganic perovskites based on $\mathrm{CsSn}I_{3}$ and $\mathrm{MASn}I_{3}$ achieve high efficiency close to their Shockley–Queisser theoretical PCE value (33.7%). These perovskites can be an alternative to improve stability, reduce resistance to degradation, reduce costs, and minimize recombination of the generated carriers [6,7].

In addition, scientists are exploring the field of perovskite solar cells with different configurations. Some alternatives involve the replacement of or variations in electron and hole transport layers (HTLs and ETLs), the substitution of cations in the perovskite layer, and the use of intermediate or outer layers in the cells to achieve higher efficiency and improve the spectral response. Improving the utilization of the incident solar spectrum may be an approach to increasing the performance of perovskite solar cells because they currently have problems with their stability due to humidity and oxygen in the environment [8]. Existing silicon and perovskite solar cells can utilize only a fraction of the solar radiation, ranging from the visible to the near infrared. In other words, the ultraviolet (UV) regions are not used and are considered harmful [9]. As a result, the first requirement for choosing a down-conversion energy material is that its absorption should be in the ultraviolet UV range and should not overlap with the absorption of perovskite. The down-conversion effect can be produced by materials such as lanthanides and non-lanthanides [10]. Although these materials produce the down-conversion effect, they are difficult to fabricate or too expensive because they are rare earths. An alternative to solve spectral mismatch losses, improve stability, and increase efficiency is to apply a silicon-rich oxide ($\mathrm{Si}O_{x}$) layer on a perovskite solar cell. This material is a silicon oxide out of stichometry that contains nanoislands and nanocrystals due to the excess silicon embedded in a silicon dioxide matrix and that emits red light when illuminated with ultraviolet UV radiation [11]. In addition, previous studies have shown that using a $\mathrm{Si}O_{x}$ film as a top coating on silicon solar cells can improve the J-V curve and external quantum efficiency (EQE) values. In these cases, $\mathrm{Si}O_{x}$ has been obtained by LPCVD and HFCVD, resulting in efficient photoluminescence and transmittance values [11,12].

In this work, an inverted perovskite solar cell, ITO/PEDOT:PSS/${\mathrm{CH}}_{3}NH_{3}\mathrm{Pb}I_{3}$/PCBM/Ag, was numerically simulated and analyzed. Parameters such as perovskite thickness, defect densities, series and shunt resistances, and operating temperature were optimized for this device, reaching an efficiency of 18.33%. Then, a new device model was proposed $\mathrm{Si}O_{x}$/ITO/PEDOT:PSS/${\mathrm{CH}}_{3}NH_{3}\mathrm{Pb}I_{3}$/PCBM/Ag, which includes a $\mathrm{Si}O_{x}$ layer as the down-conversion energy material, resulting in an efficiency increase of 22.46%. Furthermore, the photovoltaic characteristics of the experimentally obtained $\mathrm{Si}O_{x}$ were analyzed and corroborated for application in perovskite solar cells. For the latter simulation, the current–voltage output characteristics and quantum efficiency were better than those of the device without $\mathrm{Si}O_{x}$.

## 2. Materials and Methods

### 2.1. Device Structure

**Figure 1:**
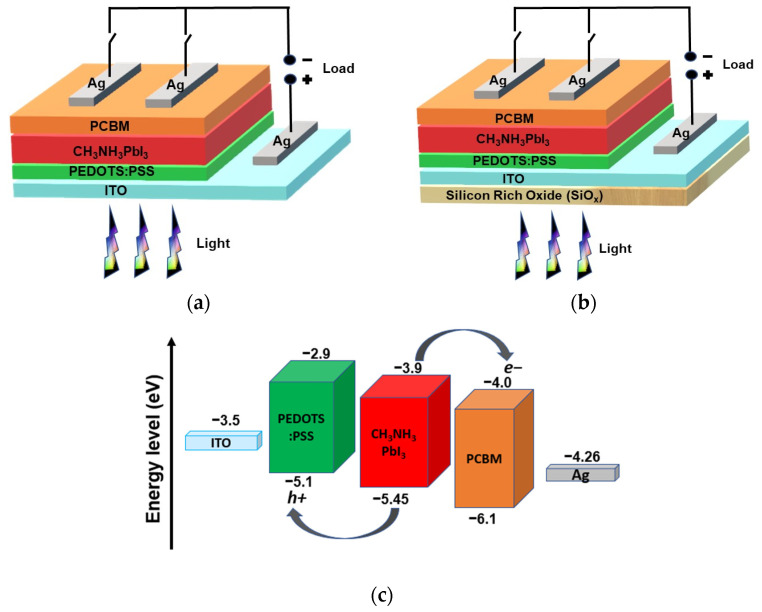
Schematic diagram of (**a**) an inverted PSC, (**b**) an inverted PSC with $\mathrm{Si}O_{x}$, and (**c**) general energy band diagram.

The structure of the simulated inverted planar perovskite solar cell has the configuration ITO/PEDOT:PSS/${\mathrm{CH}}_{3}NH_{3}\mathrm{Pb}I_{3}$/PCBM/Ag, where silver (Ag) is the back metal contact; Phenyl-C61-butyric acid and methyl ester (PCBM) form the electron transporting layer (ETL); Methylammonium Lead Iodide (${\mathrm{CH}}_{3}NH_{3}\mathrm{Pb}I_{3}$) is the perovskite absorber layer; poly(3,4-ethylenedioxythiophene) polystyrene sulfonate (PEDOT:PSS) is the hole transporting layer (HTL); and indium-doped tin oxide (ITO) is the front contact [13]. Meanwhile, the proposed $\mathrm{Si}O_{x}$/ITO/PEDOT:PSS/${\mathrm{CH}}_{3}NH_{3}\mathrm{Pb}I_{3}$/PCBM/Ag model is a device that includes a silicon-rich oxide $(\mathrm{Si}O_{x})$ layer as the down-conversion energy material on the outside of the cell. The two structures of the inverted perovskite solar device are shown in Figure 1a,b, and the energy band diagram is shown in Figure 1c. The energy band diagram proposed provides the validation of the model as the band gap of each layer is perfectly aligned for the light to reach the absorber and for charge extraction and transfer to occur in the solar cell.

### 2.2. Numerical Method

The simulation was performed in the SCAPS-1D software version 3.3.10 under AM1.5G illumination and at an ambient room temperature of 300 K. The SCAPS-1D software, developed by Professor Marc Burgelman of the Department of Electronics and Information Systems (ELIS) at the University of Ghent in Belgium, has been used to model and simulate perovskite solar cells [14]. The software solves the semiconductor equations in one dimension in a steady state [15,16]. The main equations are the Poisson Equation (1), the electron continuity Equation (2), the hole continuity Equation (3), the electron charge transport Equation (4), the hole charge transport Equation (5), and the absorption coefficient (6), which are solved until convergence occurs [17].

(1) $$\frac{\mathrm{dE}}{dx}=\frac{d^{2}\phi \left(x\right)}{dx^{2}}=\frac{q}{\varepsilon_{0}\varepsilon_{r}}\left[p\left(x\right)\;-\;n\left(x\right)+N_{D}^{+}\left(x\right)\;-\;N_{A}^{-}\left(x\right)+p_{t}\left(x\right)\;-\;n_{t}\left(x\right)\right]$$

where E is the electric field, ϕ is the electrostatic potential, q is the electron charge, $\varepsilon_{0}$ is the permittivity of vacuum, $\varepsilon_{r}$ is relative permittivity, $N_{D}^{+}$ is the ionized donor concentration, $N_{A}^{-}$ is the ionized acceptor density, and $n\left(x\right)$ and $p\left(x\right)$ are the densities of electrons and holes. In addition, $p_{t}\left(x\right)$ y $n_{t}\left(x\right)$ represents the trapped holes and electrons as a function of x.

(2) $$\frac{dJ_{n}}{\mathrm{dx}}=G\;-\;U_{n}$$

(3) $$\frac{dJ_{p}}{\mathrm{dx}}=G\;-\;U_{p}$$

G is the carrier generation rate, $U_{n}$ and $U_{p}$ are the recombination rates for electrons and holes, and $J_{n}$ and $J_{p}$ are electron and hole current densities, respectively.

(4) $$J_{n}=D_{n}\frac{\mathrm{dn}}{\mathrm{dx}}+\mu_{n}n\frac{d\phi }{\mathrm{dx}}$$

(5) $$J_{p}=D_{p}\frac{\mathrm{dp}}{\mathrm{dx}}+\mu_{p}p\frac{d\phi }{\mathrm{dx}}$$

$D_{n}$ and $D_{p}\;$are the electron and hole diffusion coefficients, and $\mu_{n\;}$and $\mu_{p}$ are the electron and hole mobility, respectively.

(6) $$\alpha (\lambda )=(A+\frac{B}{h\nu })\sqrt{h\nu \;-\;E_{g}}$$

A and B are constants, h is the Planck constant, ν is the frequency of photons, and $E_{g}$ is the band gap of the absorber layer.

### 2.3. Simulation Parameters

The physical, optical, and electrical parameters were obtained from a review of the scientific literature and are summarized in Table 1. The parameters used for the silicon-rich silicon oxide $(\mathrm{Si}O_{x})$ layer correspond to a $\mathrm{Si}O_{2}$ layer. Nevertheless, we experimentally obtained the layer thickness, photoluminescence, transmittance, absorption coefficient, and band gap for simulation purposes. Subsequently, Table 2 shows the parameters for interface defect densities and the electrical parameters of the metallic back contact.

## 3. Results and Discussion

### 3.1. Absorption Coefficient of Perovskite Layer

**Figure 2:**
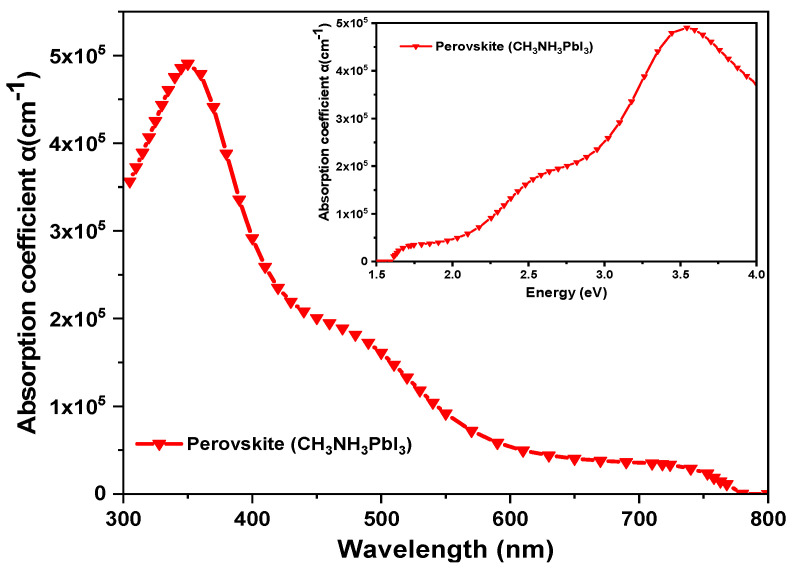
The absorption coefficient of the perovskite active layer simulated in SCAPS-1D.

The simulated absorption coefficient of the perovskite active layer was obtained from SCAPS-1D software. We extracted the data and plotted them in Figure 2. We can observe that the absorption is higher in the short wavelength region (300 nm to 500 nm) and decreases at long wavelengths (600 nm to 800 nm) until it drops to zero.

### 3.2. Effect of Perovskite Layer Thickness

Optimal selection of perovskite layer thickness is necessary to obtain a solar cell with efficient output values [35]. According to the literature, a thin perovskite layer is not advantageous due to weak light absorption, resulting in deficient short-circuit current density ($J_{\mathrm{sc}}$) and power conversion energy (PCE) values. Similarly, a large thickness is not beneficial because there will be a significant path required to transfer the photo-generated charge carriers, resulting in a weaker electric field, reduced carrier diffusion length, and a high carrier recombination rate [36]. The decrease in the open-circuit voltage $\left(V_{\mathrm{oc}}\right)\;$and PCE after the perovskite layer thickness has reached the optimum value is due to the increase in the saturation current density $\left(J_{0}\right)$, which increases the recombination of charge carriers. The dependence of open-circuit voltage $\left(V_{\mathrm{oc}}\right)\;$on the photo-generated current and the saturation current density $\left(J_{0}\right)$ is explained by Equation (7) below [37].

(7) $$V_{\mathrm{oc}}=\frac{\mathrm{KT}}{q}\ln(\frac{J_{\mathrm{sc}}}{J_{0}}+1)$$

where $\frac{\mathrm{KT}}{q}$ is the thermal voltage, $J_{\mathrm{sc}}$ is the photo-generated current density, and $J_{0}$ is the saturation current density.

**Figure 3:**
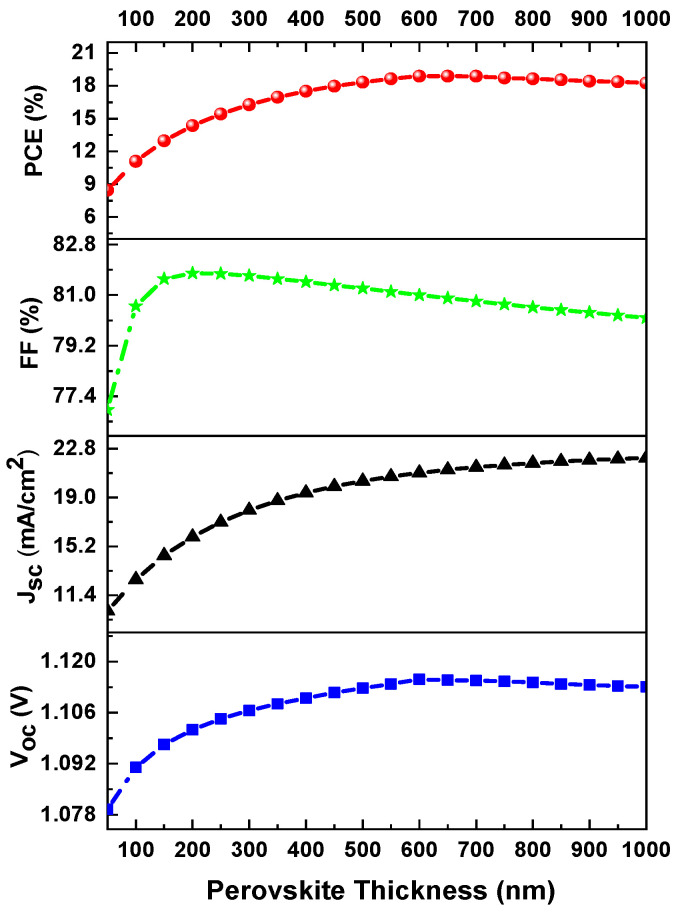
Photovoltaic characteristics of PCE, FF, $J_{\mathrm{sc}}$, and $V_{\mathrm{oc}}$ as a function of perovskite layer thickness.

Figure 3 shows the responses of PCE, FF, $J_{\mathrm{sc}}$, and $V_{\mathrm{oc}}$ as a function of the perovskite layer thickness. The variation was from 50 nm to 1000 nm, and all other parameters in Table 1 remained constant in the simulation. Furthermore, in Figure 3, $V_{\mathrm{oc}}$ initially increases from 1.078 V to 1.115 V as the thickness increases from 0 to 600 nm and then saturates and tends to decrease slightly at higher values of perovskite thickness. The decrease in $V_{\mathrm{oc}}$ with thickness is due to the increment in saturation current density. Meanwhile, $J_{\mathrm{sc}}$ increases rapidly from 9.7 $\mathrm{mA}/{\mathrm{cm}}^{2}$ to 21.5 $\mathrm{mA}/{\mathrm{cm}}^{2}$ over a thickness range from 50 nm to 1000 nm. The increase in $J_{\mathrm{sc}}$ is due to a higher rate of charge carrier generation. Moreover, FF decreases from 81.9% to 80% in the 200 nm to 1000 nm thickness range, which is due to an increase in series resistance and internal power dissipation in the thick absorber layer, while PCE steadily increases in the thickness range from 0 to 500 nm, obtaining an efficiency of 18.33%, but then starts to decrease slightly in the range from 600 to 1000 nm. Therefore, an absorber layer between 500 and 600 nm is optimal for improving the efficiency of inverted perovskite solar cells.

### 3.3. Effect of Perovskite Layer Defect Density and Interface Defects

The total defect density ${(N}_{t})$ in the perovskite absorber plays a significant role in the efficiency of the solar cell because defects create a transition energy level between the valence and conduction bands functioning as recombination centers in the perovskite layer. A high recombination rate is associated with the decrement in diffusion length and lifetime of carriers, resulting in decay in cell performance [7,28]. Recombination is a process in which electron–hole pairs are annihilated, resulting in low values of open circuit voltage ($V_{\mathrm{oc}}$), fill factor (FF), and power conversion energy (PCE). Maintaining a controlled deposition process of the perovskite layer avoids impurities and the production of deep traps. Controlling the morphology and deposition process reduces the $N_{t}$ value. Therefore, it is advisable to consider low defect densities to obtain high output values [25,26]. The defect density is derived from the Shockley–Read–Hall (SRH) recombination model and is described by Equation (8) [38].

(8) $$R_{\mathrm{SRH}}=\frac{\mathrm{np}-{n_{i}}^{2}}{\tau_{p}\left(n+N_{c}e^{\left(\frac{E_{g}-E_{t}}{\mathrm{kT}}\right)}\right)+\tau_{n}\left(p+N_{v}e^{\left(\frac{E_{t}}{\mathrm{kT}}\right)}\right)}$$

where $n_{i}$ is the charge density of the intrinsic carrier, n and p represent the electron and hole concentrations, respectively, $E_{t}$ is the energy level of the defect density, and $\tau_{n,p}$ denotes the lifetime of charges carriers.

Consequently, the minority carrier lifetime $\tau_{n,p}\;$and the carrier diffusion length $L_{D}$ are expressed by Equations (9) and (10), respectively [39].

(9) $$\tau_{n,p}=\frac{1}{N_{t}\sigma_{n,p}v_{\mathrm{th}}}$$

(10) $$L_{D}=\sqrt{\frac{\mu_{\left(n,\;p\right)}\mathrm{kT}}{q}\tau_{n,p}}$$

where $N_{t}$ is the total defect density, $\sigma_{n,p}$ is the capture cross-sectional area for electrons and holes, $v_{\mathrm{th}}$ is the thermal velocity of mobile carriers, and $\mu_{\left(n,\;p\right)}$ is the mobility of electrons and holes. Equations (8) and (9) demonstrate that if the value of $N_{t}$ increases, the carrier lifetime $\tau_{n,p}$ decreases. As a result, both $L_{D}$ and $V_{\mathrm{oc}}$ decrease. Therefore, to achieve better performance, the $N_{t}$ value of the absorber layer should have a low value [40].

**Figure 4:**
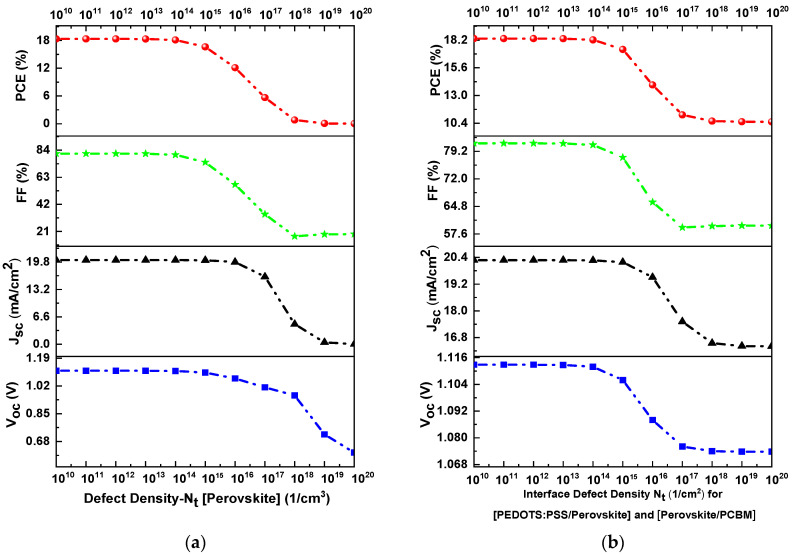
Photovoltaic characteristics of PCE, FF, $J_{\mathrm{sc}}$, and $V_{\mathrm{oc}}$ as a function of defect density of the (**a**) perovskite layer and (**b**) HTL/perovskite and perovskite/ETL interfaces.

Figure 4a shows the response of PCE, FF, $J_{\mathrm{sc}}$, and $V_{\mathrm{oc}}$ as a function of the defect density of the absorber layer. The variation in $N_{t}$ was from${\;10}^{10}$ to ${\;10}^{20}$ ${\mathrm{cm}}^{-3}$ when considering all other parameters constant in the simulation. Figure 4a shows a decrease in PCE, FF, $J_{\mathrm{sc}}$, and $V_{\mathrm{oc}}$ values as $N_{t}$ increases. Consequently, $V_{\mathrm{oc}}$ decreased from 1.10 V to 0.59 V, $J_{\mathrm{sc}}$ decreased from 19.82 $\mathrm{mA}/{\mathrm{cm}}^{2}$ to 0.09 $\mathrm{mA}/{\mathrm{cm}}^{2}$, FF decreased from 83.9% to 18%, and PCE decreased from 18.33% to 0.032% when $N_{t}$ was in the range of $10^{14}$ to $10^{20}{\;\mathrm{cm}}^{-3}$. The optimal value defect density for the perovskite layer is when it has a value of $10^{13}{\;\mathrm{cm}}^{-3}$ [26,28].

On the other hand, the density of interface defects also plays an essential role in the efficiency of the solar cell due to defects functioning as recombination sites degrading the J-V and QE (%) output results [41]. Interface defects are generally due to chemical impurities, surface dislocations, uncoordinated atoms, and dangling bonds formed on the perovskite surface in the fabrication process [42]. Figure 4b shows the responses of PCE, FF, $J_{\mathrm{sc}}$, and $V_{\mathrm{oc}}\;$as a function of the defect density of the interfaces (HTL/perovskite and perovskite/ETL). In addition, the variation in $N_{t}$ was from${\;10}^{10}$ to $10^{20}$ ${\mathrm{cm}}^{-2}$, keeping the other parameters constant in the simulation. As a result, the PCE, FF, $J_{\mathrm{sc}}$, and $V_{\mathrm{oc}}\;$values tend to decrease when $N_{t}$ varies from $10^{14}$ to $10^{20}\;{\mathrm{cm}}^{-2}$. As the defect density of the interfaces increases, all the J-V characteristics decrease [43]. For example, $V_{\mathrm{oc}}$ decreases from 1.12 V to 1.072 V, $J_{\mathrm{sc}}$ decreases from 20.2 $\mathrm{mA}/{\mathrm{cm}}^{2}$ to 16.4 $\mathrm{mA}/{\mathrm{cm}}^{2}$, FF decreases from 81% to 57.9% and PCE decreases from 18.33% to 10.4%. If the $N_{t}$ value is below $10^{15}$ ${\mathrm{cm}}^{-2}$, higher J-V values are obtained. Therefore, the optimal value of $N_{t}$ of interfaces is $10^{13}$ ${\mathrm{cm}}^{-2}$ [42,43].

### 3.4. Effect of Series Resistance $R_{\mathrm{series}}$ and Shunt Resistance $R_{\mathrm{shunt}}$

Series and shunt resistance ($R_{\mathrm{series}}$ and $R_{\mathrm{shunt}}$) also influence the efficiency of the solar cell as they determine the shape and slope of the J-V curve. The origin of the $R_{\mathrm{series}}$ is mainly associated with the contacts (ITO and silver) and electrical dissipation occurring in the perovskite and the hole and electron transport layers (HTL and ETL). Meanwhile, the origin of the $R_{\mathrm{shunt}}$ is associated with the manifestation of various recombination paths, device design, and defects introduced in the deposition process of the layers. According to the literature, low values of series resistance and high values of shunt resistance allow better performing solar cells [44]. To understand the effect of $R_{\mathrm{series}}$ and $R_{\mathrm{shunt}}$ on the performance of the perovskite solar cell, Equation (11) of the ideal diode model was used [45].

(11) $$J=J_{L}-J_{0}\left[e^{\left[\frac{q(V+J*R_{\mathrm{serie}})}{\mathrm{AkT}}\right]}-1\right]-\frac{V+J*R_{\mathrm{serie}}}{R_{\mathrm{shunt}}}$$

In an open-circuit state (when J≈0 $\mathrm{mA}/{\mathrm{cm}}^{2}$), the variables $V_{\mathrm{oc}}$ and $R_{\mathrm{shunt}}$ form the following relationship (Equation (12)).

(12) $$R_{\mathrm{shunt}}=\frac{V_{\mathrm{oc}}}{J_{L}-J_{0}\left[e^{\left[\frac{q(V_{\mathrm{oc}})}{\mathrm{AkT}}\right]}-1\right]}$$

where J is the current through the external circuit, $J_{L}$ is the light-induced current density, $J_{0}$ is the saturation current density, V is the output voltage, A is the ideality factor, k is Boltzmann constant, T is the temperature, and q is the electron charge. Consequently, low $R_{\mathrm{shunt}}$ causes a loss of photovoltage and can also affect the collected photocurrent, while a high $R_{\mathrm{series}}$ value mainly affects the FF and $J_{\mathrm{sc}}$ values [44].

**Figure 5:**
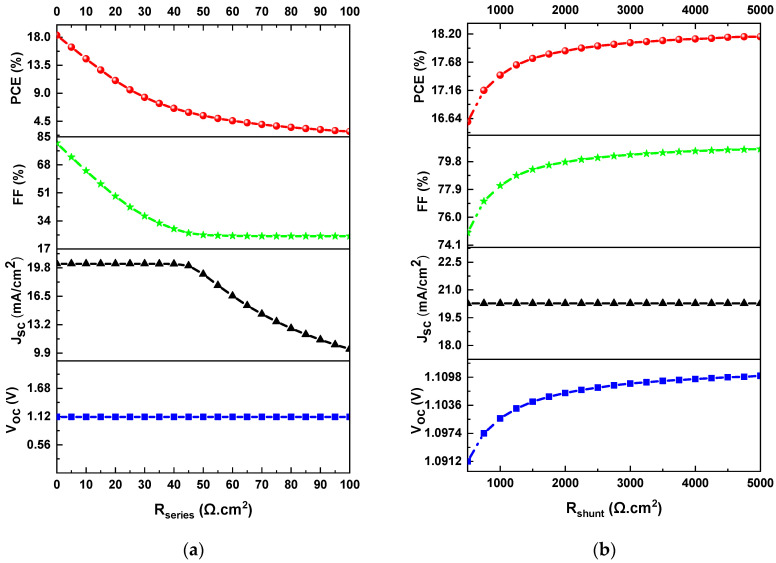
Photovoltaic characteristics of PCE, FF, $J_{\mathrm{sc}}$, and $V_{\mathrm{oc}}$ as a function of (**a**) series resistance and (**b**) shunt resistance.

To understand the effects of $R_{\mathrm{series}}$ and $R_{\mathrm{shunt}}$ on the J-V curves, these were varied from 0 to 100 $\Omega \cdot {\mathrm{cm}}^{2}\;$and from 500 to 5000 $\Omega \cdot {\mathrm{cm}}^{2}$, respectively, keeping the other parameters constant in the simulation. Figure 5a,b shows the responses of PCE, FF, $J_{\mathrm{sc}}$, and $V_{\mathrm{oc}}\;$as a function of $R_{\mathrm{series}}$ and $R_{\mathrm{shunt}}$. When $R_{\mathrm{series}}$ increases from 0 to 100 $\Omega \cdot {\mathrm{cm}}^{2}$, $V_{\mathrm{oc}}\;$remains constant, $J_{\mathrm{sc}}$ decreases from 19.82 $\mathrm{mA}/{\mathrm{cm}}^{2}$ to 9.9 $\mathrm{mA}/{\mathrm{cm}}^{2}$, and FF decreases from 80% to 25.5%. Consequently, the behavior of PCE is directly proportional to $J_{\mathrm{sc}}$ and FF, so it also decreases from 18.3% to 2.25% for the same range, as shown in Figure 5a. On the other hand, when $R_{\mathrm{shunt}}$ increases from 500 $\Omega \cdot {\mathrm{cm}}^{2}$ to 5000 $\Omega \cdot {\mathrm{cm}}^{2}$, $V_{\mathrm{oc}}$ increases from 1.091 V to 1.098 V, $J_{\mathrm{sc}}$ remains constant, FF increases from 75% to 81%, and PCE increases from 16.62% to 18.2%, as shown in Figure 5b. As a result, the optimal values for $R_{\mathrm{series}}$ and $R_{\mathrm{shunt}}$ are 3 $\Omega \cdot {\mathrm{cm}}^{2}$ and 4500 $\Omega \cdot {\mathrm{cm}}^{2}$, respectively.

### 3.5. Effect of Operating Temperature on Device Characterization

Operating temperature plays an essential role in the efficiency of a solar cell. Under installation conditions, a solar cell is subjected to temperatures higher than 300 K. Although theoretical simulation analyses show that at a temperature of 300 K, the solar cell has the best performance [46,47]. The increase in temperature causes stresses and deformations at the interfaces that consequently increase the defect density ($N_{t}$), leading to the creation of recombination centers and a reduction in diffusion length [6]. These factors directly affect the PCE and FF. The increase in temperature also causes a reduction in the semiconductor energy band gap, so the photocurrent and $J_{\mathrm{sc}}$ increase due to the decrease in recombination and the generation of more charge carriers at the interfaces. Equation (13) shows the relationship between $V_{\mathrm{oc}}$, T, and $E_{g}$ of perovskite in a solar cell [7].

(13) $$\frac{d(V_{\mathrm{oc}})}{\mathrm{dT}}=\frac{V_{\mathrm{oc}}-\;E_{g}}{T}$$

where T is the operating temperature, $E_{g}$ is the band gap, and q is the electric charge. The rate of change of $V_{\mathrm{oc}}$ is inversely proportional to temperature.

**Figure 6:**
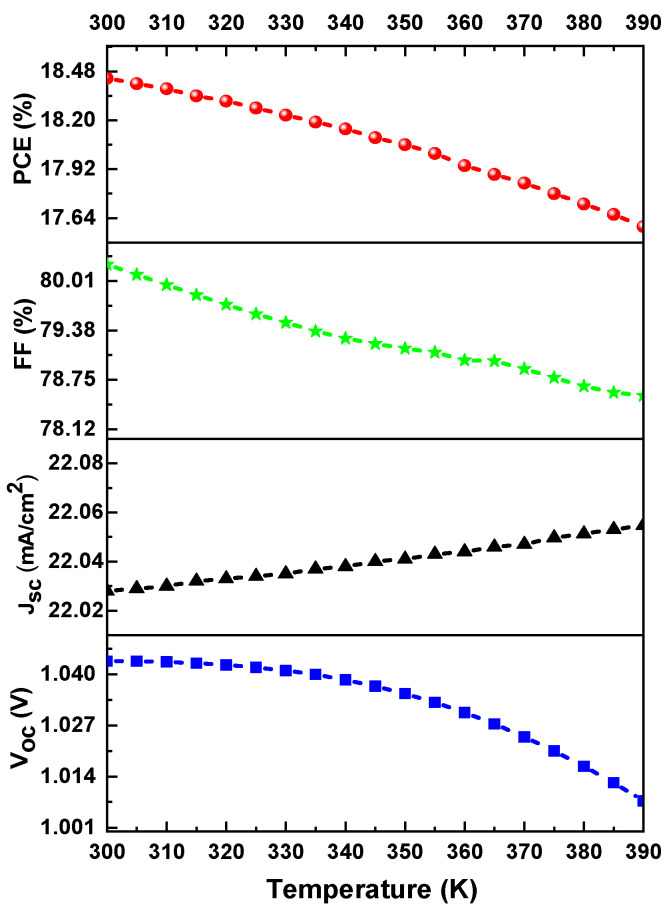
Photovoltaic characteristics of PCE, FF, $J_{\mathrm{sc}}$, and $V_{\mathrm{oc}}\;$as a function of the operating temperature of the device.

Figure 6 shows the responses of PCE, FF, $J_{\mathrm{sc}}$, and $V_{\mathrm{oc}}$ as a function of operating temperature in the solar cell. The temperature was varied from 300 K to 390 K, maintaining all other parameters in the simulation constant. As a result, $V_{\mathrm{oc}}$ decreases in value from 1.04 V to 1.02 V, FF decreases from 80.05% to 78.95%, $J_{\mathrm{sc}}$ slightly increases from 20.02 to 20.05 $\mathrm{mA}/{\mathrm{cm}}^{2}$, and PCE decreases in value from 18.4% to 17.5% as the temperature increased from 300 K to 390 K. Therefore, a temperature of 300 K is always considered optimum.

### 3.6. Photovoltaic Properties of Silicon-Rich Oxide ($\mathrm{Si}O_{x}$)

According to the literature, a down-conversion energy material absorbs high-energy photons (ultraviolet UV light) and subsequently emits low-energy photons (visible light), where a solar device has the most advantages and sensitivity. The main characteristics of a down-conversion energy material are high PL photoluminescence in the visible spectrum, broadband absorption in the region where the solar cell response spectrum is low, a high absorption coefficient in the low wavelength region, high transmittance, broadband emission where the device response is high, chemical, and environmental stability, low roughness and finally ease of processing [9,10]. In this section, the properties of the experimentally obtained silicon-rich oxide ($\mathrm{Si}O_{x}$) are presented and the main characteristics of a down-conversion energy material were determined. The $\mathrm{Si}O_{x}$ layers were deposited by co-sputtering RF using Si (2″, 99.99% purity) and $\mathrm{Si}O_{2}$ (2″, 99.99% purity) targets with a Torr International magnetron sputtering system (13.56 MHz). The RF power applied to the silicon target was 45 W, while the RF power applied to the $\mathrm{Si}O_{2}$ target was constant at 100 W. The layers were deposited on 1 inch × 1 inch × 1 mm quartz substrates. Before $\mathrm{Si}O_{x}$ deposition, the quartz substrates were cleaned in an ultrasonic bath with xylene, acetone, and deionized water. In addition, we have considered the best deposition parameters to obtain a medium silicon excess (of 5.2 at.%) and efficient photoluminescence emission intensity in the red–blue region before and after thermal annealing [48,49]. The thicknesses of the silicon-rich oxide $\mathrm{Si}O_{x}$ layers were obtained by profilometry with the Vecco Dektak 150 equipment. The thicknesses obtained were 90 nm, 88 nm, and 92 nm for three samples after thermal annealing.

**Figure 7:**
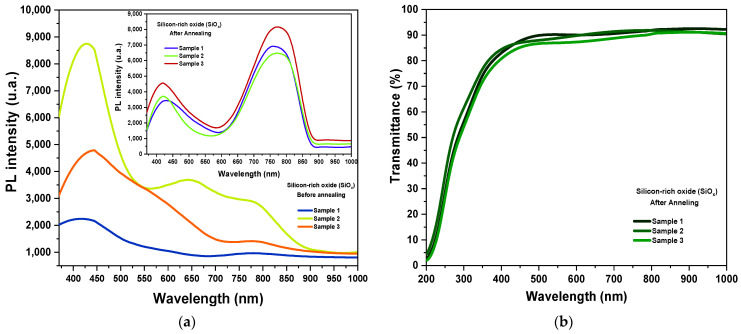
(**a**) PL spectra of $\mathrm{Si}O_{x}$ layers before and after thermal annealing and (**b**) transmittance spectra of $\mathrm{Si}O_{x}$ layers after thermal annealing.

Photoluminescence spectra were obtained using a Horiba Jobin Yvon NanoLog FR3 device and are shown in Figure 7a. This figure shows the PL photoluminescence spectra obtained for the $\mathrm{Si}O_{x}$ layers before and after thermal annealing. The PL spectra after thermal annealing show two emission bands: a higher intensity red emission band in the range of 625 nm to 875 nm with an emission peak centered at 775 nm and a second lower intensity blue emission band in the range of 375 nm to 525 nm with an emission peak centered at 425 nm. However, the spectra before thermal annealing only show a large emission band in the blue region in a range from 375 nm to 575 nm with an emission peak centered at 425 nm. The two main mechanisms associated with photoluminescence emission in $\mathrm{Si}O_{x}$ layers correspond to quantum confinement effects in silicon nanocrystals (Si-ncs) and defects, such as defects at the $\mathrm{Si}/\mathrm{Si}O_{x}$ interface and defects associated with oxygen vacancies [50,51].

Subsequently, transmittance spectra were obtained using a Varian Cary 5000 device and are shown in Figure 7b. This figure shows the transmittance spectrum of $\mathrm{Si}O_{x}$ layers after thermal annealing. $\mathrm{Si}O_{x}$ has a high transmittance of 90% in the range from 450 to 900 nm (visible and infrared spectrum), while for lower lengths between 200 and 400 nm (ultraviolet region), the transmittance drops to zero. This property is crucial as it corresponds to high transmittance and broadband emission in the region where the response of the solar device is the highest. That is, it allows visible light to enter the active layer.

The transmittance spectra of the $\mathrm{Si}O_{x}$ layers allowed us to obtain the absorption coefficient and the band gap energy $\left(E_{g}\right)$. The absorption coefficients α(λ) were determined from the Beer–Lambert law described by Equation (14) below [52].

(14) $$\alpha \;(\lambda )\;=\frac{-\ln\left[T(\lambda )\right]}{d}$$

where T(λ) is the transmittance, and d is the thickness of the $\mathrm{Si}O_{x}$ layers.

**Figure 8:**
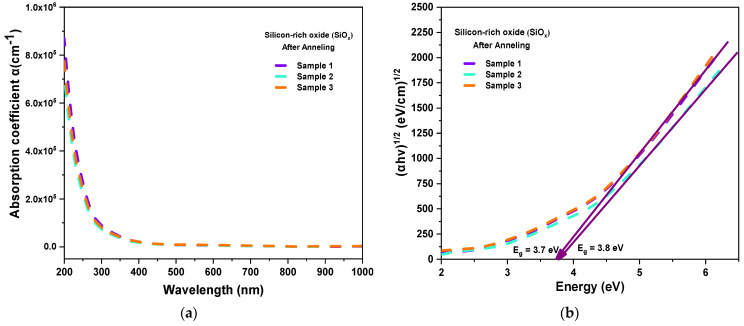
(**a**) Absorption coefficients and (**b**)${\left(\alpha \mathrm{hv}\right)}^{1/2}\;$vs. photon energy (hv) for $\mathrm{Si}O_{x}$ layers.

Figure 8a shows the absorption coefficients of the $\mathrm{Si}O_{x}$ layers. Figure 8a shows that $\mathrm{Si}O_{x}$ has higher absorption in the ultraviolet region (200 to 370 nm) and low absorption in the visible region (370 nm to 800 nm). This property is important because it corroborates that the material has a high absorption coefficient in the short wavelength region (ultraviolet light), where the response of the solar cell is low, so the implementation of this layer can contribute to improved stability.

To determine the band gap energy, we used the equation known as the Tauc plot, which is described by Equation (15) below [53].

(15) $${\left(\alpha \mathrm{hv}\right)}^{1/2}=A(\mathrm{hv}-E_{g})$$

where α is the absorption coefficient, (hv) is the photon energy, $E_{g}$ is the band gap energy, and A is a constant. From a plot ${\left(\alpha \mathrm{hv}\right)}^{1/n}$ versus hv, the band gap can be extrapolated from a straight line to hv =0. We used n=1/2, which indicates that an indirect optical transition is allowed for this material [54].

Figure 8b shows the method used for calculating the energy band gap ${(E}_{g})$. The $E_{g}$ values obtained from the $\mathrm{Si}O_{x}$ layers are 3.7 eV, 3.7 eV, and 3.8 eV. Therefore, the thickness, absorption coefficient, and $E_{g}$ of $\mathrm{Si}O_{x}$, were edited in SCAPS-1D to improve the output responses of the inverted perovskite solar cell.

### 3.7. Simulated J-V Characteristics Curve, External Quantum Efficiency, and Energy Band Diagram

**Figure 9:**
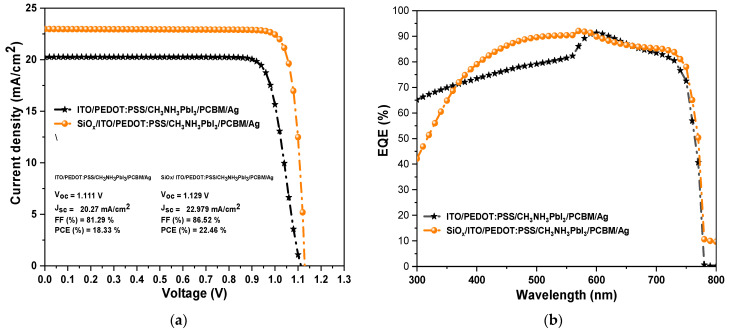
(**a**) Current density–voltage curve and (**b**) EQE of ITO/PEDOT: PSS/${\mathrm{CH}}_{3}NH_{3}\mathrm{Pb}I_{3}$/PCBM/Ag and $\mathrm{Si}O_{x}$/ITO/PEDOT:PSS/${\mathrm{CH}}_{3}NH_{3}\mathrm{Pb}I_{3}$/PCBM/Ag solar cells.

The ITO/PEDOT:PSS/${\mathrm{CH}}_{3}NH_{3}\mathrm{Pb}I_{3}$/PCBM/Ag solar device and the $\mathrm{Si}O_{x}$/ITO/PEDOT:PSS/${\mathrm{CH}}_{3}NH_{3}\mathrm{Pb}I_{3}$/PCBM/Ag solar device were simulated in SCAPS-1D and the final characteristic curve of current density and voltage for both devices are plotted in Figure 9a. In addition, the optimal values obtained from the simulation were considered for both devices. The optimal values considered were a perovskite thickness of 500 nm, a perovskite defect density of $10^{13}$ ${\mathrm{cm}}^{-3}$, an interface defect density of $10^{13}$ ${\mathrm{cm}}^{-2}$, a series and shunt resistance of 3 $\Omega \cdot {\mathrm{cm}}^{2}$ and 4500 $\Omega \cdot {\mathrm{cm}}^{2}$, respectively, and an operating temperature of 300 K. For the $\mathrm{Si}O_{x}\;$solar device, the experimentally obtained parameters, such as a thickness of 90 nm, a band gap of 3.8 eV, and the absorption coefficient obtained from the transmittance, were considered. As a result, the inverted perovskite solar cell ITO/PEDOT:PSS/${\mathrm{CH}}_{3}NH_{3}\mathrm{Pb}I_{3}$/PCBM/Ag produced output values of $V_{\mathrm{oc}}=1.111\;V$, $J_{\mathrm{sc}}=20.27\;\mathrm{mA}/{\mathrm{cm}}^{2}$, FF=81.29%, and PCE=18.33%, while the $\mathrm{Si}O_{x}$/ITO/PEDOT:PSS/${\mathrm{CH}}_{3}NH_{3}\mathrm{Pb}I_{3}$/PCBM/Ag inverted perovskite solar cell produced output values of $V_{\mathrm{oc}}=1.129\;V$, $J_{\mathrm{sc}}=22.979\;\mathrm{mA}/{\mathrm{cm}}^{2}$, FF=86.52%, and PCE=22.46%.

Subsequently, we obtained the EQE (external quantum efficiency) or IPCE (incident-photon-to-current efficiency) for both devices (Figure 9b). EQE is a parameter to evaluate the quality of a perovskite solar cell and is often used to demonstrate the spectral response of incident photons. The quantum efficiency is the ratio of the total charge carriers extracted from the cell to the total number of incident photons. For the numerical analysis of EQE, the simulation was performed at wavelengths between 300 and 800 nm for both devices. We can observe that the ITO/PEDOT:PSS/${\mathrm{CH}}_{3}NH_{3}\mathrm{Pb}I_{3}$/PCBM/Ag device shows an average EQE of 75% between 500 nm and 580 nm and subsequently shows an average EQE of 85% between 580 nm and 750 nm, with a maximum peak of 91% at 600 nm. Furthermore, the proposed $\mathrm{Si}O_{x}$/ITO/PEDOT: PSS/${\mathrm{CH}}_{3}NH_{3}\mathrm{Pb}I_{3}$/PCBM/Ag device shows an average EQE of 87% between 400 nm and 750 nm (generally in the visible spectrum), with a maximum peak of 92% at 570 nm. Therefore, the increase in the EQE of the proposed solar device occurs at short wavelengths, where there is more absorption of the perovskite film, and also due to the photovoltaic parameters of the $\mathrm{Si}O_{x}$ material included in the simulation ($E_{g}$, PL, absorption coefficient, and thickness).

**Figure 10:**
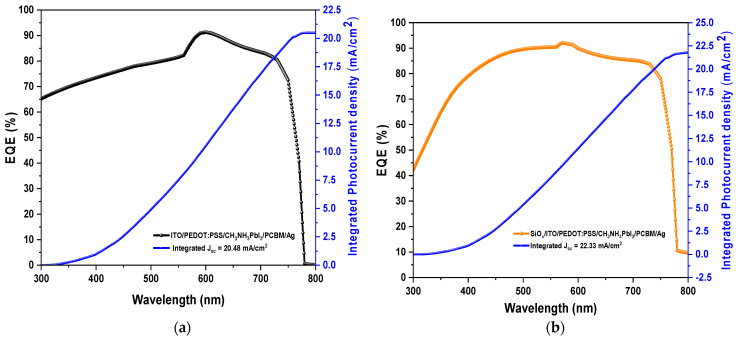
EQE spectrum and integrated photocurrent density $J_{\mathrm{sc}}$ (blue line): (**a**) for device ITO/PEDOT:PSS/${\mathrm{CH}}_{3}NH_{3}\mathrm{Pb}I_{3}$/PCBM/Ag; and (**b**) for device $\mathrm{Si}O_{x}$/ITO/PEDOT:PSS/${\mathrm{CH}}_{3}NH_{3}\mathrm{Pb}I_{3}$/PCBM/Ag.

Afterward, we obtained the value of the integrated photocurrent density ($J_{\mathrm{sc}}$) from the EQE data of both devices (Figure 10a,b). The exact $J_{\mathrm{sc}}$ value was calculated with the integral of the product of EQE and the incident photon flux over the spectral distribution. We obtained values of $J_{\mathrm{sc}}=20.48\;\mathrm{mA}/{\mathrm{cm}}^{2}$ and $J_{\mathrm{sc}}=22.33\;\mathrm{mA}/{\mathrm{cm}}^{2}$ for the ITO/PEDOT:PSS/${\mathrm{CH}}_{3}NH_{3}\mathrm{Pb}I_{3}$/PCBM/Ag and $\mathrm{Si}O_{x}$/ITO/PEDOT:PSS/${\mathrm{CH}}_{3}NH_{3}\mathrm{Pb}I_{3}$/PCBM/Ag devices, respectively. These values are very similar to those obtained for the two devices from the J-V curves shown previously in Figure 9a. In addition, they indicate the maximum photogenerated current density that can be obtained in each device.

**Figure 11:**
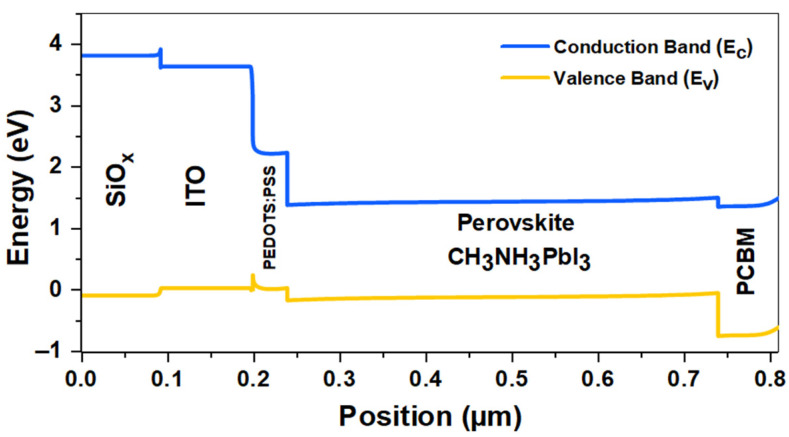
Simulated energy band diagram of the $\mathrm{Si}O_{x}$/ITO/PEDOT:PSS/${\mathrm{CH}}_{3}NH_{3}\mathrm{Pb}I_{3}$/PCBM/Ag solar cell.

In addition, the SCAPS-1D software allows us to obtain the energy band diagram of the solar cell. Figure 11 shows the energy band diagram of the final inverted perovskite solar cell whose structure is $\mathrm{Si}O_{x}$/ITO/PEDOT:PSS/${\mathrm{CH}}_{3}NH_{3}\mathrm{Pb}I_{3}$/PCBM/Ag. There is a splitting of the valence band and conduction band, as well as an increase in the thickness of the perovskite layer due to optimization. The diagram indicates that the PEDOT:PSS and PCBM layers have higher energy band gaps concerning the perovskite layer, so there is a band alignment that favors the passage of electrons and holes generated in the perovskite. Meanwhile, energy barriers at the interfaces prevent minority carrier transport from the perovskite to the HTL and ETL transport layers. In addition, the $\mathrm{Si}O_{x}$ layer enables affinity, band alignment, and enhanced photon transmission to the absorber, which contributes to higher electron–hole pair generation, so more current is generated. Finally, the improved current flow leads to increased efficiency.

### 3.8. Comparison between Experiment and Simulation

Table 3 shows the reported PCE, FF, $J_{\mathrm{sc}}$, and $V_{\mathrm{oc}}$ values for inverted perovskite solar cells with methylammonium lead iodide perovskite $\left({\mathrm{CH}}_{3}NH_{3}\mathrm{Pb}I_{3}\right)$. In addition, the devices were selected because they share a similarity with the one proposed in this work. Thus, we performed a comparative analysis with the results obtained from the simulation using the optimized parameters. The results obtained in this work match with those reported experimentally. Subsequently, when we add the $\mathrm{Si}O_{x}$ layer, a 4.13% increase in power conversion efficiency (PCE) is observed. Hence, the $\mathrm{Si}O_{x}$/ITO/PEDOT: PSS/${\mathrm{CH}}_{3}NH_{3}\mathrm{Pb}I_{3}$/PCBM/Ag device could be an alternative to improve the performance and stability of inverted perovskite solar cells. Our results are the first experimental and simulation comparison reported to date.

## 4. Conclusions

In this work, we numerically simulated and optimized an inverted planar perovskite solar cell with ITO/PEDOT:PSS/${\mathrm{CH}}_{3}NH_{3}\mathrm{Pb}I_{3}$/PCBM/Ag structure using SCAPS-1D. First, we analyzed the effects of varying the thickness of the absorber layer, defect density at the absorber layer and interfaces, series and shunt resistance, and operating temperature on the device. The simulation results suggest that an absorber layer thickness of 500 nm, perovskite defect density of $10^{13}{\;\mathrm{cm}}^{-3}$, interface defect density of $10^{13}{\;\mathrm{cm}}^{-2}$, series and shunt resistance of 3 $\Omega \cdot {\mathrm{cm}}^{2}$ and 4500 $\Omega \cdot {\mathrm{cm}}^{2}$, respectively, and an operating temperature of 300 K improve the current density–voltage curve. Secondly, we compared the simulation results with the experimental reports, obtaining a similar results for the PCE, FF, $J_{\mathrm{sc}}$, and $V_{\mathrm{oc}}$ parameters. Finally, a $\mathrm{Si}O_{x}$ layer was added to the structure as a down-conversion energy material to obtain the $\mathrm{Si}O_{x}$/ITO/PEDOT:PSS/${\mathrm{CH}}_{3}NH_{3}\mathrm{Pb}I_{3}$/PCBM/Ag device which produced better output values in the J-V and EQE (%) plots. Therefore, the $\mathrm{Si}O_{x}$ layer was implemented with a thickness of 90 nm, an $E_{g}$ of 3.8 eV, and its absorption coefficient in the simulation, yielding values of $V_{\mathrm{oc}}=1.12\;V$, $J_{\mathrm{sc}}=22.97\;\mathrm{mA}/{\mathrm{cm}}^{2}$, FF=86.5%, and PCE=22.46%.

In conclusion, the silicon-rich oxide $\left(\mathrm{Si}O_{x}\right)$ material obtained by RF co-sputtering satisfies the main characteristics indicated in the literature of a down-conversion energy material for organic and perovskite solar cell applications. Moreover, there are no previous experimental reports or simulations of inverted perovskite solar cells containing $\mathrm{Si}O_{x}$ in their structure. Therefore, our study could provide a basis for the alternative design and fabrication of cost-effective, efficient, and stable inverted perovskite solar cells.

## Figures and Tables

**Table 1:** Optical and electrical parameters used for simulation.

Parameters	$\mathrm{Si}O_{x}$ [18]	ITO [19,20,21]	PEDOT:PSS (HTL) [22,23,24]	Perovskite CH_3_HN_3_PbI_3_ [25,26,27]	PCBM (ETL) [28,29,30]
Thickness (nm)	90	100	40	300	80
Band gap, $E_{g}\;(\mathrm{eV})$	3.8	3.5	2.2	1.55	2.1
Electron affinity, χ (eV)	0.950	2.3	2.9	3.9	4.1
Relative permittivity, ε	3.9	9	2.3	18	4
Effective CB density of states, $N_{c}\;(1/{\mathrm{cm}}^{3})$	$2.8\;\times {\;10}^{18}$	$2.2\;\times {\;10}^{18}$	$2.2\;\times {\;10}^{18}$	$2.2\;\times {\;10}^{18}$	$2.5\;\times {\;10}^{19}$
Effective VB density of states, $N_{v}\;(1/{\mathrm{cm}}^{3})$	$2.6\;\times {\;10}^{19}$	$1.8\;\times {\;10}^{19}$	$2.8\;\times {\;10}^{18}$	$1.9\;\times {\;10}^{19}$	$2.5\;\times {\;10}^{19}$
Electron thermal velocity, $V_{n}\;(\mathrm{cm}/s)\;$	$1\;\times {\;10}^{7}$	$1\;\times {\;10}^{7}$	$1\;\times {\;10}^{7}$	$1\;\times {\;10}^{7}$	$1\;\times {\;10}^{7}$
Hole thermal velocity, $V_{p}\;(\mathrm{cm}/s)\;$	$1\;\times {\;10}^{7}$	$1\;\times {\;10}^{7}$	$1\;\times {\;10}^{7}$	$1\;\times {\;10}^{7}$	$1\;\times {\;10}^{7}$
Electron mobility, $\mu_{n}\;({\mathrm{cm}}^{2}/\mathrm{Vs})$	$1.5\;\times {\;10}^{3}$	31	0.0002	3	0.01
Hole mobility, $\mu_{p}\;({\mathrm{cm}}^{2}/\mathrm{Vs})$	$4.5\;\times {\;10}^{3}$	50	0.02	17	0.01
Donor concentration, $N_{D}\;(1/{\mathrm{cm}}^{3})$	0	0	0	$1\;\times {\;10}^{15}$	$5\;\times {\;10}^{17}$
Acceptor concentration, $N_{A}\;(1/{\mathrm{cm}}^{3})$	$2\;\times {\;10}^{18}$	$2\;\times {\;10}^{16}$	$1\;\times {\;10}^{18}$	$1\;\times {\;10}^{15}$	0
Defect density, $N_{t}\;(1/{\mathrm{cm}}^{3})$	${\;10}^{15}$	${\;10}^{15}$	${\;10}^{15}$	${\;10}^{17}$	${\;10}^{15}$

**Table 2:** Simulation parameters for defects and metal contact.

**Parameters**	**CH_3_HN_3_PbI_3_** **[25,31,32]**	**HTL/Perovskite** **[25]**	**Perovskite/ETL** **[25]**
Defect type Electron capture cross section $\;({\mathrm{cm}}^{2})$ Hole capture cross section $\;({\mathrm{cm}}^{2})$ Energetic distribution Reference for defect energy level $\;E_{t}$ Energy level with respect to reference (eV) Total density (integrated over all energies) $\;(1/{\mathrm{cm}}^{3})$	Neutral $\begin{array}{c} 1\;\times {\;10}^{-15}\\ 1\;\times {\;10}^{-15} \end{array}$ Gaussian Below $\;E_{c}$ 0.65 $10^{17}$	Neutral $\begin{array}{c} 1\;\times {\;10}^{-15}\\ 1\;\times {\;10}^{-15} \end{array}$ Single Above $\;E_{V}$ 0.6 $1\;\times \;10^{15}$	Neutral $\begin{array}{c} 1\;\times {\;10}^{-15}\\ 1\;\times {\;10}^{-15} \end{array}$ Single Above $\;E_{V}$ 0.6 $1\;\times \;10^{15}$
**Parameters**			**Silver (Ag)** **[33,34]**
Work function (eV) Surface recombination velocity of electrons (cm/s) Surface recombination velocity of holes (cm/s)			4.26 $\begin{array}{c} 10^{7}\\ {\;10}^{5} \end{array}$

**Table 3:** Comparative analysis with experimental data.

Device Structure	$\begin{array}{c} V_{\mathrm{oc}}\\ \left(V\right) \end{array}$	$\begin{array}{c} J_{\mathrm{sc}}\\ (\mathrm{mA}/{\mathrm{cm}}^{2}) \end{array}$	$\begin{array}{c} \mathrm{FF}\\ \left(\%\right) \end{array}$	$\begin{array}{c} \mathrm{PCE}\\ \left(\%\right) \end{array}$	Ref.
ITO/PEDOT:PSS/CH_3_NH_3_PbI_3_/PCBM/Ca/Al (experimental)	1.03	20.6	85	18	[55]
ITO/PEDOT:PSS/CH_3_NH_3_PbI_3_/PCBM/Au (experimental)	1.1	20.9	83	18.1	[56]
ITO/DMF-PEDOT:PSS/CH_3_NH_3_PbI_3_/PCBM/BCP/Ag (experimental)	1.02	22.38	82	18.72	[13]
ITO/PEDOT:PSS/CH_3_NH_3_PbI_3_/PCBM/Ag (simulated)	1.11	20.27	81.29	18.33	This work
$\mathrm{Si}O_{x}$/ITO/PEDOT:PSS/CH_3_NH_3_PbI_3_/PCBM/Ag (simulated)	1.12	22.97	86.52	22.46	This work

## Data Availability

Data is contained within the article.

## References

[1] Chao L., Niu T., Gao W., Ran C., Song L., Chen Y., Huang W. (2021). Solvent Engineering of the Precursor Solution toward Large-Area Production of Perovskite Solar Cells. Adv. Mater..

[2] Yang J., Luo X., Zhou Y., Li Y., Qui Q., Xie T. (2022). Recent Advances in Inverted Perovskite Solar Cells: Designing and Fabrication. Int. J. Mol. Sci..

[3] Yin Z., Lu B., Chen Y., Guo C. (2022). Advances of Commercial and Biological Materials for Electron Transport Layers in Biological Applications. Front. Bioeng. Biotechnol..

[4] Mazumdar S., Zhao Y., Zhang X. (2021). Stability of Perovskite Solar Cells: Degradation Mechanisms and Remedies. Front. Electron..

[5] Mohanty I., Mangal S., Jana S., Singh U.P. (2021). Stability factors of perovskite (CH_3_NH_3_PbI_3_) thinfilms for solar cell applications: A study. Mater. Today Proc..

[6] Imani S., Seyed-Talebi S.M., Beheshtian J., Guang Diau E.W. (2023). Simulation and characterization of CH_3_NH_3_SnI_3_-based perovskite solar cells with different Cu-based hole transporting layers. Appl. Phys. A.

[7] Karna L.R., Upadhyay R., Ghosh A. (2023). All-inorganic perovskite photovoltaics for power conversion efficiency of 31%. Sci. Rep..

[8] Jiang L., Chen W., Zheng J., Zhu L., Mo L., Li Z., Hu L., Hayat T., Alsaedi A., Zhang C. (2017). Enhancing the Photovoltaic Performance of Perovskite Solar Cells with a Down-Conversion Eu-Complex. ACS Appl. Mater. Interface.

[9] Datt R., Bishnoi S., Lee H.K.H., Arya S., Gupta S., Gupta V., Tsoi W.C. (2022). Down-conversion materials for organic solar cells: Progress, challenges, and perspectives. Aggregate.

[10] Datt R., Bishnoi S., Hughes D., Mahajan P., Singh A., Gupta R., Arya S., Gupta V., Tsoi W.C. (2022). Downconversion Materials for Perovskite Solar Cells. Sol. RRL.

[11] Vivaldo I., Carrillo J., López O., Jiménez S., Martínez J., Murias D., López J.A. (2016). Study of the photon down-conversion effect produced by thin silicon-rich oxide films on silicon solar cells. Int. J. Energy Res..

[12] Ojeda-Durán E., Monfil-Leyva K., Carrillo-López J., Benítez-Lara A., García-Salgado G., Luna-López J.A. (2019). Down-Conversion Effect Created by SiOx Films Obtained by HFCVD and Applied over Pn-Junctions. Silicon.

[13] Chen K., Hu Q., Liu T., Zhao L., Luo D., Wu J., Zhang Y., Zhang W., Liu F., Russel T.P. (2016). Charge-Carrier Balance for Highly Efficient Inverted Planar Heterojunction Perovskite Solar Cells. Adv. Mater..

[14] Burgelman M., Nollet P., Degrave S. (2000). Modelling polycrystalline semiconductor solar cells. Thin Solid Films.

[15] Verschraegen J., Burgelman M. (2007). Numerical modeling of intra-band tunneling for heterojunction solar cells in scaps. Thin Solid Films.

[16] Burgelman M., Decock K., Kheli S., Abass A. (2013). Advanced electrical simulation of thin film solar cells. Thin Solid Films.

[17] Burgelman M., Decock K., Niemegeers A., Verschraegen J., Degrave S. (2021). SCAPS Manual.

[18] Ghosh B., Nasir S., Chee F., Routray S., Saad I., Mohamad K. (2022). Numerical study of nSi and nSiGe solar cells: Emerging microstructure nSiGe cell achieved the highest 8.55% efficiency. Opt. Mater..

[19] Ghosh A., Dipta S., Nikor S., Saqib N., Saha A. (2020). Performance analysis of an efficient and stable perovskite solar cell and a comparative study of incorporating metal oxide transport layers. J. Opt. Soc. Am. B.

[20] Umar A., Sadanand, Singh P.K., Dwivedi D.K., Algadi H., Ibrahim A.A., Alhammai M.A.M., Baskoutas S. (2022). High Power-Conversion Efficiency of Lead-Free Perovskite Solar Cells: A Theoretical Investigation. Micromachines.

[21] Lakhdar N., Hima A. (2020). Electron transport material effect on performance of perovskite solar cells based on CH_3_NH_3_GeI_3_. Opt. Mater..

[22] Sabbah H., Arayro J., Mezher R. (2022). Simulation and Investigation of 26% Efficient and Robust Inverted Planar Perovskite Solar Cells Based on GA_0.2_FA_0.78_SnI_3_-1%EDAI_2_ Films. Nanomaterials.

[23] Ahamed T., Rahaman I., Karmakar S., Halim M.A., Sarkar P.K. (2023). Thickness optimization and the effect of different hole transport materials on methylammonium tin iodide (CH_3_NH_3_SnI_3_)-based perovskite solar cell. Emergent Mater..

[24] Gan Y., Bi X., Liu Y., Qin B., Li Q., Jiang Q., Mo P. (2020). Numerical investigation energy conversion performance of tin-based perovskite solar cells using cell capacitance simulator. Energies.

[25] Basyoni M.S.S., Salah M.M., Mousa M., Shaker A., Zekry A., Abouelatta M., Alshammari M.T., Al-Dhlan K.A., Gontrand C. (2021). On the Investigation of Interface Defects of Solar Cells: Lead-Based *vs.* Lead-Free Perovskite. IEEE Access.

[26] Ouslimane T., Et-taya L., Elmaimouni L., Benami A. (2021). Impact of absorber layer thickness, defect density, and operating temperature on the performance of MAPbI_3_ solar cells based on ZnO electron transporting material. Heliyon.

[27] Gholami-Milani A., Ahmadi-Kandjani S., Olyaeefar B., Kermani M.H. (2023). Performance analyses of highly efficient inverted all-perovskite bilayer solar cell. Sci. Rep..

[28] Jamal M.S., Shahahmadi S.A., Wadi M.A.A., Chelvanathan P., Asim N., Misran H., Hossain M.I., Amin N., Sopian K., Akhtaruzzaman M. (2019). Effect of defect density and energy level mismatch on the performance of perovskite solar cells by numerical simulation. Optik.

[29] Roy P., Sinha N.K., Khare A. (2020). An investigation on the impact of temperature variation over the performance of tin-based perovskite solar cell: A numerical simulation approach. Mater. Today Proc..

[30] Shamna M.S., Nithya K.S., Sudheer K.S. (2020). Simulation and optimization of CH_3_NH_3_SnI_3_ based inverted perovskite solar cell with NiO as Hole transport material. Mater. Today Proc..

[31] Rana A., Sharma P., Kumar A., Pareek S., Waheed S., Singh R.K., Karak S. (2021). Understanding the Correlation Between Temperature Dependent Performance and Trap Distribution for Nickel Oxide Based Inverted Perovskite Solar Cells. IEEE Trans. Electron Devices.

[32] Kumar A., Rana A., Vashistha N., Garg K.K., Singh R.K. (2020). Defect states influencing hysteresis and performance of perovskite solar cells. Sol. Energy.

[33] Abdy H., Aletayeb A., Kolahdouz M., Soleimani E.A. (2019). Investigation of metal-nickel oxide contacts used for perovskite solar cell. AIP Adv..

[34] Wang J., Li J., Zhou Y., Yu C., Hua Y., Yu Y., Li R., Lin X., Chen R., Wu H. (2021). Tuning an Electrode Work Function Using Organometallic Complexes in Inverted Perovskite Solar Cells. J. Am. Chem. Soc..

[35] Isoe W., Mageto M., Maghanga C., Mwamburi M., Odari V., Awino C. (2020). Thickness Dependence of Window Layer on CH_3_NH_3_PbI_3-x_Cl_x_ Perovskite Solar Cell. Int. J. Photoenergy.

[36] Zhu H., Balaban A.T., Klein D.J., Zivkovic T.P. (2020). Performance optimization of CH_3_NH_3_Pb(I_1-x_Br_x_)_3_ based perovskite solar cells by comparing different ETL materials through conduction band offset engineering. Opt. Mater..

[37] Singh P., Rivindra N.M. (2012). Temperature dependence of solar cell performance-an analysis. Mater. Sol. Cells.

[38] Devi C., Mehra R. (2019). Device simulation of lead-free MASnI_3_ solar cell with CuSbS_2_ (copper antimony sulfide). J. Mater. Sci..

[39] Gan Y., Zhao D., Qin B., Bi X., Liu Y., Ning W., Yang R., Jiang Q. (2022). Numerical Simulation of High-Performance CsPbI_3_/FAPbI_3_ Heterojunction Perovskite Solar Cells. Energies.

[40] Du H.J., Wang W.C., Zhu J.Z. (2016). Device simulation of lead-free CH_3_NH_3_SnI_3_ perovskite solar cells with high efficiency. Chin. Phys. B.

[41] Lei Y., Xu Y., Wang M., Zhu G., Jin Z. (2021). Origin, Influence, and Countermeasures of Defects in Perovskite Solar Cells. Small.

[42] Haque M.M., Mahjabin S., Khan S., Hossain M.I., Muhammad G., Shahiduzzaman M., Sopian K., Akhtaruzzaman M. (2022). Study on the interface defects of eco-friendly perovskite solar cells. Sol. Energy.

[43] Mohanty I., Mangal S., Singh U.P. (2022). Influence of defect densities on perovskite (CH_3_NH_3_PbI_3_) solar Cells: Correlation of experiment and simulation. Mater. Today Proc..

[44] Karthick S., Velumani S., Bouclé J. (2020). Experimental and SCAPS simulated formamidinium perovskite solar cells: A comparison of device performance. Sol. Energy.

[45] Li Y., Ding B., Chu Q.Q., Yang Y., Wang M., Li C.X., Li C.J. (2017). Ultra-high open-circuit voltage of perovskite solar cells induced by nucleation thermodynamics on rough substrates. Sci. Rep..

[46] Piñón Reyes A.C., Ambrosio Lázaro R.C., Monfil Leyva K., Luna López J.A., Flores Méndez J., Heredia Jiménez A.H., Muñoz Zurita A.L., Severiano Carrillo F., Ojeda Durán E. (2021). Study of a Lead-Free Perovskite Solar Cell Using CZTS as HTL to Achieve a 20% PCE by SCAPS-1D Simulation. Micromachines.

[47] Medina J.C.Z., Andrés E.R., Ruíz C.M., Espinosa E.C., Yarce L.T., Galeazzi Isasmendi R., Trujillo R.R., Salgado G.G., Solis A.C., Caballero F.G.N. (2023). Numerical Simulation and Performance Optimization of a Solar Cell Based on WO_3_/CdTe Heterostructure Using NiO as HTL Layer by SCAPS 1D. Coatings.

[48] Coyopol A., Cardona M.A., Díaz Becerril T., Licea Jimenez L., Morales Sánchez A. (2016). Silicon excess and thermal annealing effects on structural and optical properties of co-sputtered SRO films. J. Lumin..

[49] Coyopol A., Cabañas-Tay S.A., Díaz Becerril T., García-Salgado G., Palacios-Huerta L., Morales-Morales F., Morales-Sánchez A. (2017). Enhancement of the luminescence by the controlled growth of silicon nanocrystals in SRO/SiO_2_ superlattices. Superlattices Microstruct..

[50] Iacona F., Franzò G., Spinella C. (2000). Correlation between luminescence and structural properties of Si nanocrystals. J. Appl. Phys..

[51] López J.A.L., López J.C., Valerdi D.E.V., Salgado G.G., Díaz Becerril T., Pedraza A.P., Gracia F.J.F. (2012). Morphological, compositional, structural, and optical properties of Si-nc embedded in SiO_x_ films. Nanoscale Res. Lett..

[52] Swinehart D. (1962). The Beer-Lambert Law. J. Chem. Educ..

[53] Tauc J., Grigorovici R., Vancu A. (1966). Optical Properties and Electronic Structure of Amorphous Germanium. Phys. Status. Soidi B.

[54] Hernández Simón Z.J., Luna López J.A., Hernández De la Luz J.A.D., Pérez García S., Benítez Lara A., García Salgado G., Carrillo López J., Mendoza Conde G.O., Martínez Hernández H.P. (2020). Spectroscopic Properties of Si-nc in SiO_x_ Films Using HFCVD. Nanomaterials.

[55] Wu C.G., Chiang C.H., Tseng Z.L., Nazeeruddin M.K., Hagfeldt A., Grätzel M. (2015). High efficiency stable inverted perovskite solar cells without current hysteresis. Energy Environ. Sci..

[56] Heo J.H., Han H.J., Kim D., Ahn T.K., Im S.H. (2015). Hysteresis-less inverted CH_3_NH_3_PbI_3_ planar perovskite hybrid solar cells with 18.1% power conversion efficiency. Energy Environ. Sci..

